# Feedback-driven event-related potentials in conditional discrimination: insights from a matching-to-sample study

**DOI:** 10.3389/fnhum.2026.1557497

**Published:** 2026-02-20

**Authors:** Kyle Joseph Edmunds, Mo-Ya Chu, Erik Arntzen, Paolo Gargiulo, Hanna Steinunn Steingrimsdottir

**Affiliations:** 1 Reykjavík University, Reykjavik, Iceland; 2 School of Medicine and Public Health, Wisconsin Alzheimer's Institute, University of Wisconsin-Madison, Madison, WI, United States; 3 Department of Behavioral Sciences, Oslo Metropolitan University, Oslo, Norway; 4 Department of Science, National University Hospital of Iceland, Reykjavik, Iceland

**Keywords:** conditional discrimination, EEG, event-related potentials, feedback, learning, matching-to-sample, stimulus equivalence

## Abstract

This study examined differences in event-related potentials (ERP) associated with the presentation of programmed consequences during conditional discrimination training in a matching-to-sample (MTS) paradigm. Electroencephalography (EEG) data were continuously recorded from n = 11 participants using a 64-channel wet-electrode system at a sampling frequency of 1,024 Hz. Three-phase MTS training and testing were customized using PsychoPy and featured 12 arbitrarily related abstract stimuli explicitly designed for this study. EEG data processing and averaging were performed using ASA-Pro v. 4.10 using 0.5–40 Hz band-pass filtration and automatic artifact detection. Time-locked epochs for ERP analyses utilized a 1,000-ms window with a 200-ms pre-stimulus baseline; epochs were synchronized with electronic trigger codes associated with incorrect or correct programmed consequences following comparison stimulus selection. The difference between incorrect and correct feedback on mean ERP amplitude was significant, *t*(10) = −2.93, *p* = 0.015, *d* = −0.88, with a mean amplitude difference of 3.89 𝜇V (95% CI: [0.93, 6.86]). The effect on mean ERP latency was also significant, *t*(10) = −5.46, *p* = 0.0003, *d* = −1.65, with a mean latency difference of 109.2 ms (95% CI: [64.7, 153.7]). ERP amplitude differences were further associated with *Phase III* test scores, *t*(10) = −3.14, *p* = 0.005, *d* = −0.95, while their association with latency differences was not significant, *t*(10) = 1.46, *p* = 0.161, *d* = 0.44. Altogether, these findings underscore the sensitivity of ERP measures to feedback presentation during an MTS paradigm, lending new insight into the cortical neurodynamics during the establishment of conditional discrimination.

## Introduction

Stimulus equivalence research aims to characterize the basic processes involved in learning the meaning of words (Dias et al., 2021; Sidman, 1994). A typical training arrangement in this field is the matching-to-sample (MTS) procedure, which involves exposing participants to a series of conditional discriminations before testing for the potential establishment of two or more distinct classes of equivalent stimuli. For example, when using a linear-series MTS training structure (Saunders and Green, 1999), a participant is first exposed to a sample stimulus (e.g., “A1”), which is followed by the presentation of three (or more) comparison stimuli, where one (e.g., “B1”) is defined as correct by the experimenter and the other two (e.g., “B2” and “B3”) as incorrect. Upon selecting a comparison stimulus, the participant is presented with programmed consequences, such as feedback on the accuracy of their selection (e.g., “correct” or “incorrect”). Once this first set of conditional discriminations is established, the previous comparison stimuli may now be presented in another set as new sample singular, stimulus with an additional set of comparison stimuli—for sample stimulus “B1”, selecting its new comparison “C1” would generate a “correct” programmed consequence, whereas “C2” or “C3” would generate “incorrect.” In typical MTS training, the participant is exposed to an equal number of presentations of each trial type within a defined training block, and repeated exposures to these conditional discriminations typically result in successfully learning each relation.

Following conditional discrimination training, participants are tested on different stimulus combinations from the baseline training that assess the defining properties of an equivalence relation: symmetry (if a*R*b and b*R*c, then b*R*a and c*R*b), transitivity (if a*R*b and b*R*c, then a*R*c), and equivalence (if a*R*b and b*R*c, then c*R*a) (Sidman and Tailby, 1982). A stimulus class is considered established if participants respond in accordance with all these equivalence relation properties.

To date, most studies on stimulus equivalence have focused on different variables influencing the overt behavior emitted by the participant (Arntzen, 2012), with “overt behavior” referring to any response that can be observed directly by two or more independent observers (Ortu, 2012). Expanding behavior analytic research to include additional measures, however, provides opportunities to investigate the mechanisms involved in learning by capturing covert behavior—responses that are not directly observable (e.g., Dickins, 2005; Dickins et al., 2001). One such measure is electroencephalography (EEG), which enables the study of cortical neurodynamics during stimulus equivalence tasks (Dias et al., 2021). EEG is a non-invasive neuroimaging method used to measure the voltage fluctuations of neurons in the underlying cortex of the brain (Bera, 2015; Hassan and Wendling, 2018). These electrical potentials are then amplified and recorded, providing a continuous representation of electrocortical activity over time. Owing to its sub-millisecond-level temporal resolution, EEG is particularly well-suited for studying the rapid dynamics of cognitive processes (Aubonnet et al., 2020; Edmunds et al., 2019).

Event-related potentials (ERPs) are electrophysiological waveforms derived by averaging EEG segments, or epochs, that are time-locked to repeatedly presented stimulus events. An example is the N400 component, which is a negative deflection that peaks around 400 milliseconds after stimulus onset that reflects the extent to which stimuli share semantic features (Bortoloti et al., 2014; Granerud-Dunvoll et al., 2019; Kutas and Hillyard, 1984; Tabullo et al., 2015). With the N400 being a well-known ERP component associated with semantic relations, it is natural that this component has been targeted by researchers studying stimulus equivalence (e.g., Barnes-Holmes et al., 2005; Dias et al., 2021; Granerud-Dunvoll et al., 2019; Wang and Kameda, 2005). Findings from these studies consistently replicate the N400 effect, demonstrating that EEG can reveal neurophysiological correlates of covert behavior during equivalence class formation and thereby advance our understanding of those processes. Building on this foundation, the present study shifts attention from the “end product” of equivalence research (the N400 as a marker of class formation) to the early phases of the MTS experimental paradigm, examining whether EEG can shed light on additional neural activity involved in the establishment of the original baseline conditional discriminations.

Successful learning depends on sensitivity to the consequences of one’s actions: repeating behaviors that produce favorable outcomes and discontinuing those that do not. In this regard, researchers have identified ERP components associated with error processing and feedback evaluation across a variety of paradigms, such as guessing task (Ruchsow et al., 2002), gambling tasks (Gehring and Willoughby, 2002; Zheng et al., 2017), time-estimation tasks (e.g., Miltner et al., 1997; Pornpattananangkul and Nusslock, 2015), and tasks based on monetary incentives (Broyd et al., 2012; Novak et al., 2016) to name a few. Notably, the type of stimuli used in these experimental tasks may vary as well, such as using visual (Novak et al., 2016; Pornpattananangkul and Nusslock, 2015; Zheng et al., 2017) or auditory stimuli (Krugliakova et al., 2018; Miltner et al., 1997). These ERPs include the error-related negativity (ERN), a response-locked component that peaks approximately 100 ms after an erroneous response (e.g., Gehring et al., 2018); the feedback-related negativity (FRN), which emerges 200–300 ms after feedback presentation (e.g., Miltner et al., 1997); and the P300, a positive deflection typically observed 300–500 ms post-stimulus (e.g., Paul et al., 2025; San Martín, 2012). Notably, there are ongoing empirical, conceptual, and methodological discussions related to the presence and interpretation of ERN, FRN, and P300 (see, for example, Krigolson, 2018; Nunez-Estupinan et al., 2022; San Martín, 2012; Walsh and Anderson, 2012). Reporting on these issues is beyond the scope of this work, but a consistent finding is that these ERP components are sensitive to feedback, with larger amplitudes generally elicited by negative outcomes (San Martín, 2012).

Since the conditional discrimination procedure is extensively used by behavior analysts to teach stimulus relations, it is of interest to expand our current knowledge of the mechanisms involved in learning. EEG offers a promising tool for examining co-occurring covert processes during training. To date, however, we are unaware of studies investigating the role of feedback-related ERPs in the establishment of conditional discriminations. Using an exploratory framework, this study employed high-density 64-channel EEG to measure differences in ERP latency and amplitude associated with the presentation of programmed consequences following correct and incorrect responses during MTS training. This work further examines whether these differences in these ERP measures were associated with equivalence class formation in the final test phase of the experimental paradigm.

## Method

### Participants

**Figure 1:**
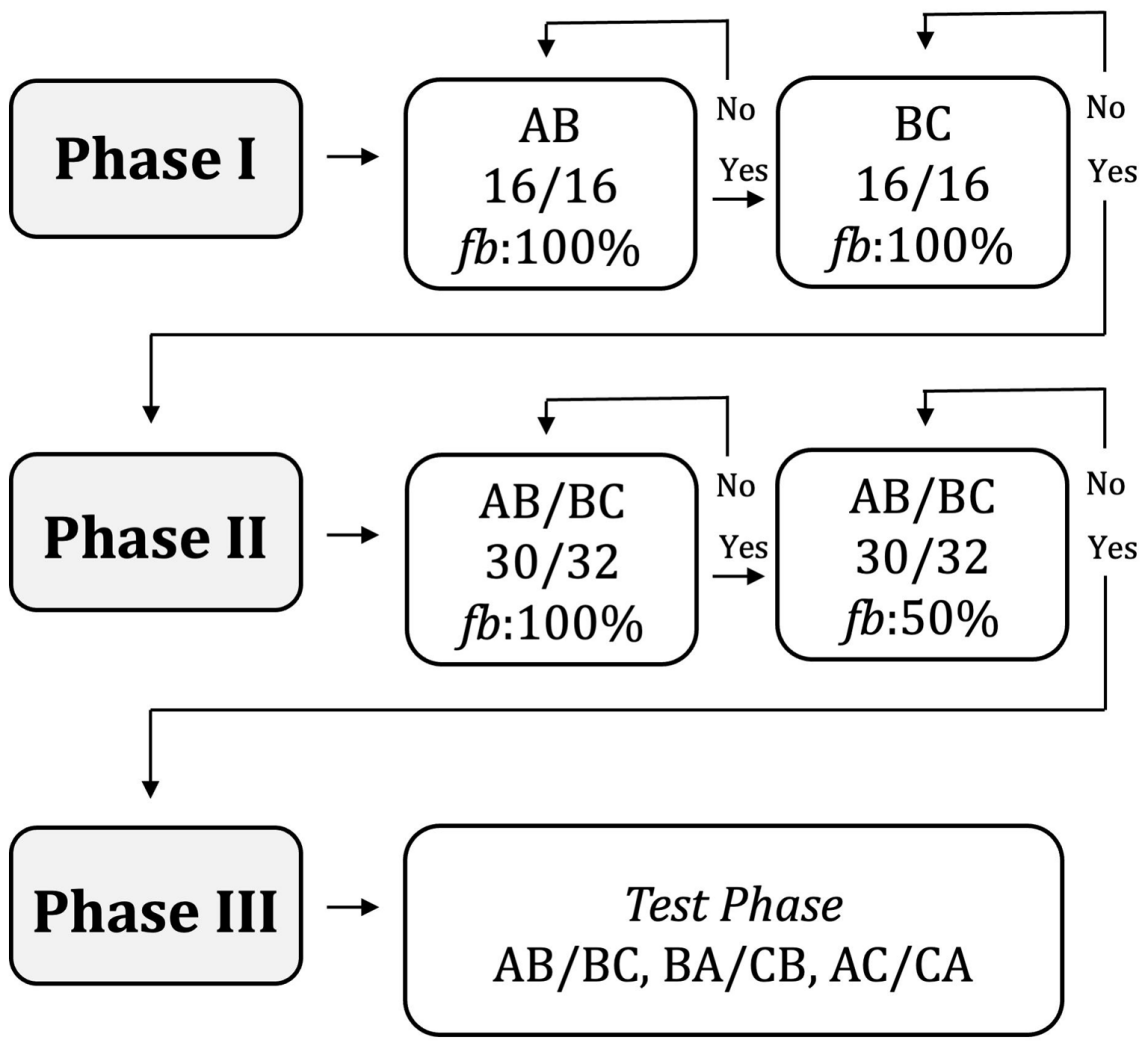
Graphical summary of the MTS experimental paradigm for this work, defined by three experimental phases: conditional discrimination training (phase I), maintenance (*Phase II*), and testing (*Phase III*). *Phase I* and *II* blocks further denote associated mastery criteria and the percent likelihood of feedback presentation.

Sixteen young adult volunteers were recruited for this study through the authors’ social networks and campus advertisements. Of these, five participants were excluded from the analyses. One participant (P13) failed to progress beyond *Phase I* (Figure 1) after more than 1 h of attempting, reported frustration, and chose to discontinue the experiment. Three participants (P03, P04, P06) experienced interruptions that required the procedure to be restarted, resulting in substantially greater learning exposure than the rest of the sample; their data were therefore excluded. Finally, one participant (P07) achieved 32 consecutive correct responses across the first two training blocks (16 in each), an outcome highly unlikely given that most participants required 64–112 trials to reach the criterion of 16 consecutive correct responses. This suggested programming or recording error, and the data were therefore excluded.

After applying these exclusion criteria, the final sample included n = 11 participants (6 male, 5 female), and their ages ranged from 20 to 29 years with a mean ± SD age of 23.1 ± 2.6 years. All participants provided written informed consent upon arrival and were briefed on the study background, data handling procedures, and their rights as research participants. The experiment lasted approximately 90–150 min for all participants.

### Experimental conditions

The MTS training for this study included four sets of conditional discriminations, each with three members. The study was segmented into three experimental phases: *Phase I*−Training, *Phase II*−Maintenance, and *Phase III*−Testing (Figure 1). During *Phase I*, four “AB” and four “BC” baseline conditional discriminations were established across two training blocks, with each response to the comparison stimulus followed by a programmed consequence. Mastery was set to 16/16 trials in each training block to proceed. *Phase II* featured a training block with an even mix of all baseline conditional discriminations (“AB and BC”) with a 30/32 mastery criterion—first with a 100% feedback probability, followed by an analogous training block with a 50% feedback probability. This prepared the participant for *Phase III*, where all combinations of stimuli within each stimulus class were presented in one testing block without any programmed consequences being presented.

### Experimental stimuli

**Figure 2:**
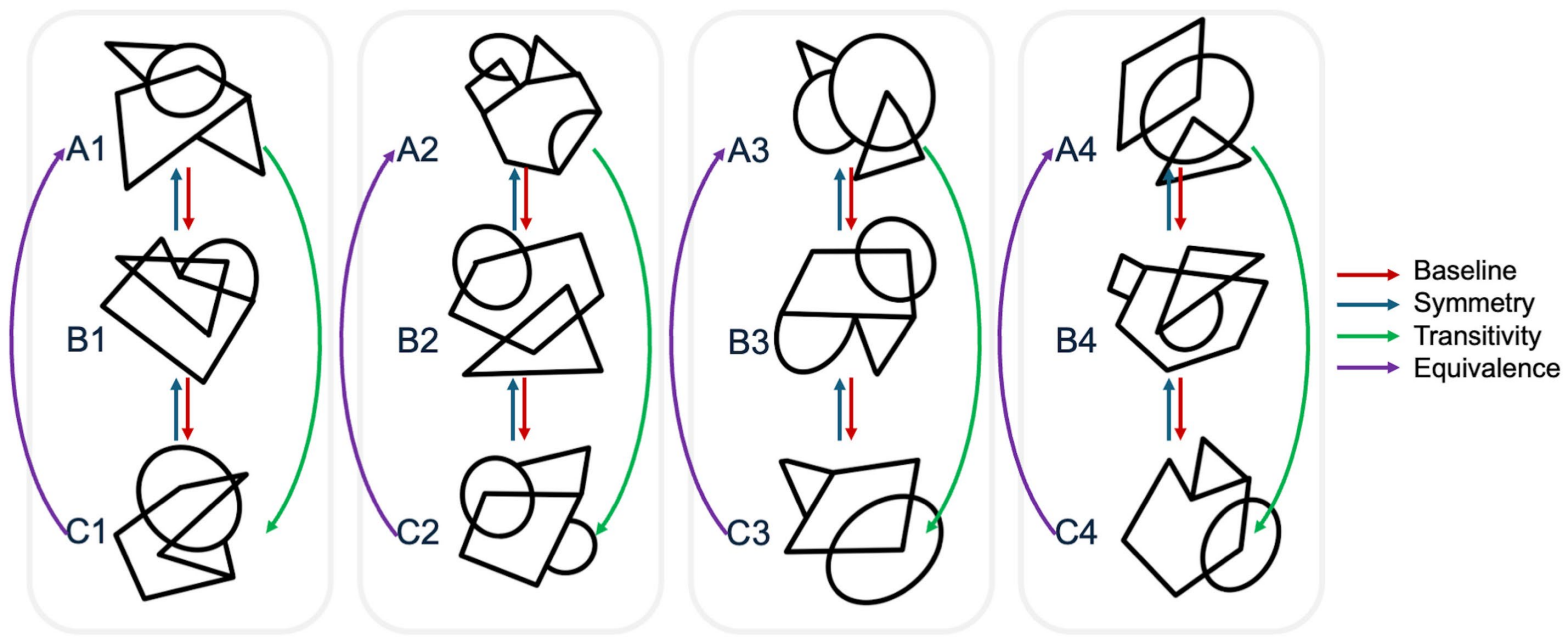
The experimental stimuli and linear-series training structure for this study.

The second author of this study created 12 arbitrarily related abstract stimuli for the experiment. Figure 2 illustrates each of these stimuli and the linear-series training structure used in this work.

### Experimental procedure

Before the experiment, informed consent was obtained, and a pre-sorting was performed, where participants were asked to sort index cards that depicted all 12 stimuli used in the study. Following sorting, each participant was prepared for a 64-channel EEG (see details below) and led to a desktop stimulus computer that implemented the MTS training and testing using a program, which was designed using PsychoPy.^1^ Participants were seated approximately 60–70 cm from the center of the stimulus monitor, which enabled them to comfortably view presented stimuli without excessive eye strain or head movement. Participants were provided with the following instructions before starting conditional discrimination training:

*‘A stimulus image will be presented at the center of the screen.*

*Click on the image to generate three comparison images, which will appear at the screen's top left, top right, and bottom right corners.*

*Click on the comparison image that corresponds to the presented stimulus.*

*You will receive feedback based on whether your choice was 'Correct' or 'Incorrect'. This feedback will gradually disappear towards the end of the experiment.*

*Do your best to stay relaxed but focused and try to get every response correct.*

*Click the Start button to continue..’*

Conditional discrimination training (visual–visual matching) followed a linear-series training structure with serialized introduction of the training trials; hence, training began with the establishment of four types of conditional discriminations: “A1B1”, “A2B2”, “A3B3”, and “A4B4” (*Phase I*). Each trial began with presenting a sample stimulus (e.g., “A1”), which appeared at the center of the stimulus monitor. Participants clicked on the sample stimulus to generate four comparison stimuli displayed in each of the four corners of the stimulus monitor (simultaneous MTS). One of the comparison stimuli was defined as “correct” (in this example, “B1”), whereas the other three were defined as “incorrect” (“B2”, “B3”, and “B4”). The participant then emitted a selection response, clicking on one of the comparison stimuli with the computer mouse. The selection response was followed by immediate presentation of programmed consequence (e.g., “correct” or “incorrect”) displayed centrally on the stimulus monitor in large text (for 1,000 ms). Finally, a blank inter-trial interval was set to 500 ms before the generation of the next sample stimulus.

Each trial type (e.g., “A1B1”) was presented randomly four times, amounting to a total of 16 training trials within each block. All training trials within each block were presented randomly to each participant, and the locations of the four comparison stimuli were likewise randomized to the four corners of the stimulus monitor. The experimental paradigm was designed to synchronize electronic trigger codes with EEG data collection (see details below). A mastery criterion for *Phase I* was set to 16/16 correct responses within each training block. If a participant provided 15 or fewer correct responses, the training block was repeated. When responding with 16/16 correct responses, the next set of conditional discrimination, e.g., “B1C1”, “B2C2”, “B3C3”, and “B4C4”, was introduced. The training arrangement was the same as for the first four conditional discriminations.

In *Phase II*, participants were exposed to a mix of all eight AB and BC conditional discriminations; with a larger training block of 32 training trials (four presentation of each conditional discrimination) the mastery criterion was set to 30/32 correct responses. If participants responded below mastery in either phase, the respective training or maintenance block was repeated until mastery was achieved. During *Phase II*, the training arrangement was identical to the previous training trials with one exception: the likelihood of programmed consequence presentation was gradually reduced from 100% likelihood to 50% and finally 0%.

Finally, *Phase III* included randomized combinations of all stimuli within each class, featuring an equal frequency of prior baseline discriminations, as well as symmetry (“BA”), transitivity (“AC”), and equivalence (“CA”) relations, which were presented in one testing block. Each test trial was presented in the same manner as the training trials, apart from the absence of programmed consequences (i.e., sample stimulus presentation was first, followed by the response to the sample, the presentation of four comparison stimuli with one defined as correct, and finally the selection response to one of the comparison stimuli—but with no programmed consequences during testing and a 500 ms blank screen inter-trial interval).

### 64-channel EEG acquisition and ERP analyses

EEG data were continuously recorded using a high-definition 64-channel wet-electrode eego™ mylab system developed by ANT Neuro (Enschede, Netherlands) at a sampling frequency of 1,024 Hz with a CPz reference electrode. The experimenters began by placing an EEG cap on the participant’s head—after which each channel was filled with a conductive electrode gel to establish an electrical impedance of less than 20 kΩ, which was monitored on a connected acquisition tablet. After this, experimenters led participants to the stimulus monitor, where they began the conditional discrimination training and testing.

EEG data processing and ERP averaging were performed using ASA-Pro (v. 4.10). First, a 0.5–40 Hz band-pass filter and a 50 Hz notch filter were used to eliminate mains artifacts. Next, automatic artifact detection was used to reject epochs contaminated by ocular or muscular activity; flagged segments were visually inspected to ensure accuracy of rejection. Time-locked epochs were defined using a 1,000-ms window with a 200-ms pre-stimulus baseline centered on trigger codes synchronized with the selection response (mouse click) that was immediately followed by the presentation of the programmed consequences. ERP waveforms were obtained by separately averaging correct and incorrect feedback epochs, and peak amplitudes and latencies were extracted as local maxima within the fixed latency window, with the electrode channel of each peak also recorded.

### Statistical analyses

Paired sample t-tests assessed the association between incorrect and correct ERP amplitudes and latencies, and Cohen’s *d* values were computed to estimate effect sizes. To further explore potential associations between feedback-related amplitude and latency differences and *Phase III* test scores, a series of *post hoc* linear regression analyses was performed. These analyses also included additional regression models to examine whether these relationships were mediated by the number of *Phase II* maintenance trials or the total number of *Phase I* and *Phase II* trials. Statistical significance was defined as *p* < 0.05 for all analyses.

## Results

### MTS training and testing

As shown in Table 1, the total number of *Phase I* training trials varied significantly among participants, ranging from 96 to 304 trials; the distribution of “AB” and “BC” trials also varied, with most participants having a higher number of “AB” trials. The proportion of correct ERPs ranged from 33.7 to 79.5%, and *Phase III* test scores ranged from 52.5 to 99.2%, together indicating considerable variability in participant performance during both training and testing. Artifacted training ERPs were generally low, ranging from 1.0 to 15.3% in 10 of the 11 participants—the exception being participant P02, where 41.8% of trial epochs were removed due to artifacts.

**Table 1:** Participant training and testing data, showing the number of *Phase I* training trials and their corresponding feedback-related ERPs for each participant ranked by their *Phase III* test score (%).

Participant	*Phase I* AB trials: *n*	*Phase I* BC trials: n	Total *Phase I* trials: *n*	Correct ERPs: *n*, (%)	Incorrect ERPs: *n*, (%)	Artifacted ERPs: *n*, (%)	*Phase III* score: %
*P10*	128	64	192	134, (69.8)	56, (29.1)	2, (1.0)	99.17
*P12*	160	128	288	143, (49.7)	140, (48.6)	5, (1.7)	99.17
*P02*	112	96	208	70, (33.7)	51, (24.5)	87, (41.8)	98.33
*P15*	128	48	176	140, (79.5)	31, (17.6)	5, (2.8)	98.33
*P14*	64	64	128	91, (71.1)	34, (26.6)	3, (2.3)	85.83
*P08*	64	32	96	64, (66.7)	20, (20.8)	12, (12.5)	76.67
*P16*	112	64	176	98, (55.7)	55, (31.3)	23, (13.1)	73.33
*P11*	64	80	144	77, (53.5)	45, (31.3)	22, (15.3)	72.50
*P01*	80	64	144	109, (75.7)	25, (17.4)	10, (6.9)	61.67
*P05*	96	32	128	77, (60.2)	45, (35.2)	6, (4.7)	57.50
*P09*	224	80	304	169, (55.6)	118, (38.8)	17, (5.6)	52.50

ERPs: Event-Related Potentials; experimental phases: *Phase I* (training), *Phase II* (maintenance), and *Phase III* (testing).

### ERP waveform amplitude and latency as a function of feedback

**Figure 3:**
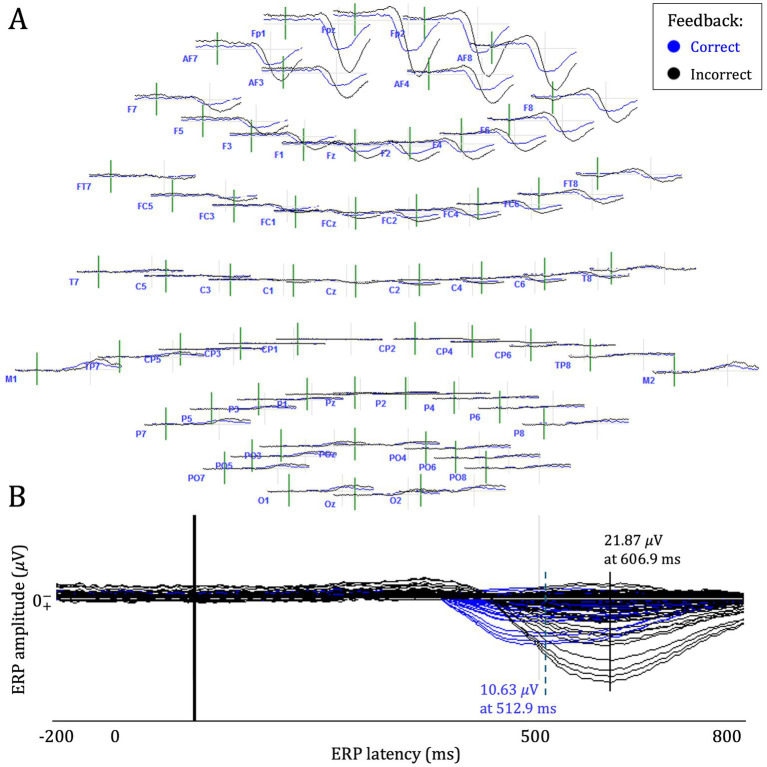
The figure shows an example of a cortical map **(A)** and ERP waveform overlay plot **(B)** from participant P05.

As evidenced in Table 2, participants exhibited higher ERP peak amplitudes following incorrect compared to correct programmed consequences, with one exception in participant P02. In contrast, ERP peak latencies were higher in incorrect epochs for all participants. Furthermore, ERP peaks were consistently recorded across frontal channels (Fp1, Fp2, FpZ, and AF7) for both conditions across all participants. Figure 3 contains an example cortical map and overlay plot of feedback-related ERP waveforms for one participant in the study.

**Table 2:** Feedback-related ERP data from *Phase I* training trials showing ERP peak amplitudes, latencies, and channel locations, as well as ERP amplitude and latency differences between correct and incorrect feedback.

Participant	Feedback	ERP peak amplitude: 𝜇V	ERP peak latency: ms	ERP peak channel(s)	∆ ERP amplitude: 𝜇V	∆ ERP latency: ms
*P01*	Correct	8.08	589.8	Fp2	8.45	161.1
	Incorrect	16.53	750.9	FpZ, Fp1		
*P02*	Correct	12.05	485.4	Fp1	−3.66	91.6
	Incorrect	8.39	577.0	FpZ, Fp1, Fp2		
*P05*	Correct	10.63	512.9	FpZ	11.24	94.0
	Incorrect	21.87	606.9	FpZ		
*P08*	Correct	9.39	798.5	FpZ, Fp1, Fp2, AF7	1.41	154.5
	Incorrect	10.80	953.0	FpZ, Fp1, Fp2, AF7		
*P09*	Correct	5.85	605.1	Fp2	1.07	28.2
	Incorrect	6.92	633.3	Fp2		
*P10*	Correct	1.11	471.7	FpZ, Fp1, AF7	0.68	138.2
	Incorrect	1.79	609.9	FpZ, Fp1, AF7		
*P11*	Correct	3.58	675.7	Fp1	6.45	141.1
	Incorrect	10.03	816.8	FpZ, Fp1		
*P12*	Correct	5.52	403.4	Fp2	2.90	242.6
	Incorrect	8.42	646.0	FpZ, Fp1, Fp2		
*P14*	Correct	2.31	154.0	Fp1, AF7	1.54	31.7
	Incorrect	3.85	185.7	AF7		
*P15*	Correct	4.22	515.6	FpZ, Fp1, Fp2	3.8	85.3
	Incorrect	8.02	600.9	FpZ, Fp1, Fp2		
*P16*	Correct	6.04	593.8	FpZ, Fp1, Fp2, AF7	8.93	32.7
	Incorrect	14.97	626.5	AF7		

ERPs: Event-Related Potentials; ∆ ERP amplitude and ∆ ERP latency: (incorrect–correct).

**Figure 4:**
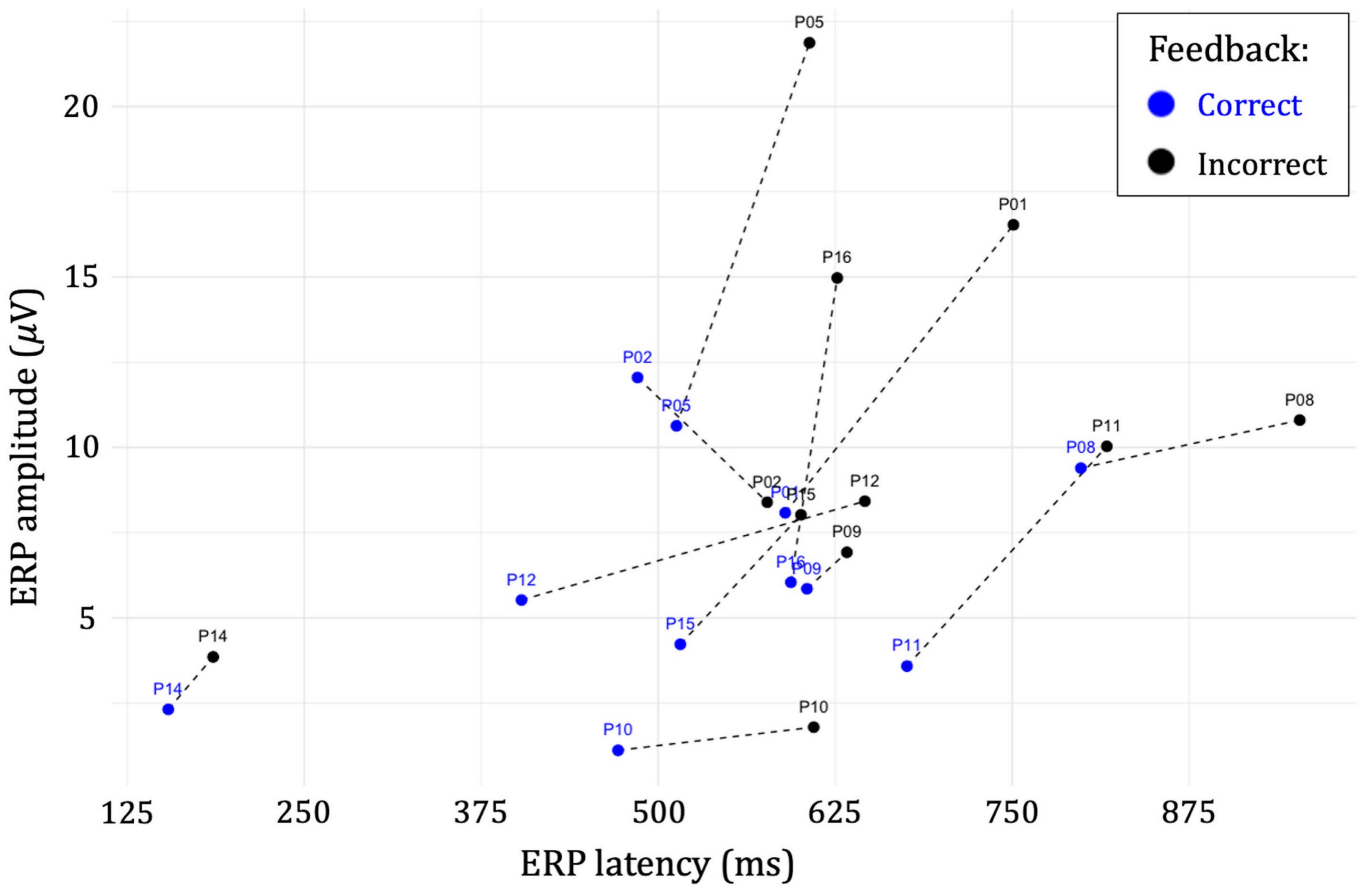
Spaghetti plot of the relationship between correct and incorrect ERP latencies and amplitudes for each of the *n* = 11 participants.

Paired sample t-tests of the difference between incorrect and correct ERP amplitudes was significant, *t* (10) = −2.93, *p* = 0.015, *d* = −0.88, indicating a difference in mean amplitude of 3.89 𝜇V (95% CI: [0.93, 6.86]) for the cohort. The effect on ERP latency was also significant, *t* (10) = −5.46, *p* = 0.0003, *d* = −1.65, indicating a significant difference in mean latency of 109.2 ms (95% CI: [64.7, 153.7]) between correct and incorrect ERP waveforms, respectively. Figure 4 shows a spaghetti plot of the relationship between correct and incorrect ERP latencies and amplitudes for each of the n = 11 study participants.

### Paired associations with *Phase III* test scores

As described, *Post hoc* linear regression analyses were performed to explore the relation between feedback-related amplitude and latency differences and *Phase III* test scores. Additional regression models were assembled to test whether these relationships were mediated by either the number of *Phase II* maintenance trials or the total number of *Phase I* and *II* trials.

**Figure 5:**
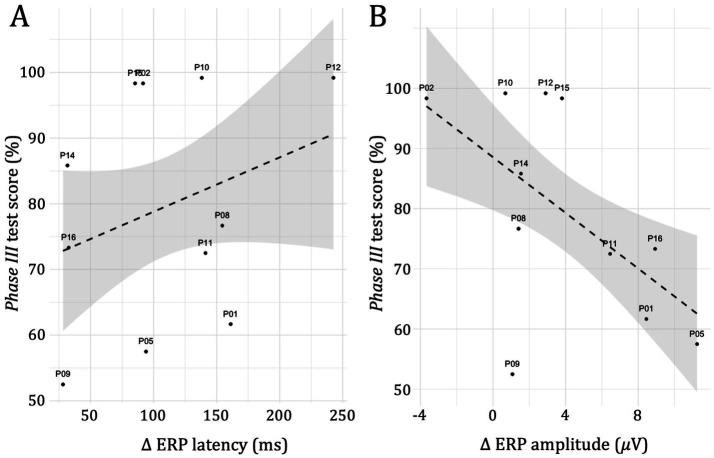
Regression plots of *Phase III* test scores show linear associations and 95% CIs between **(A)** ERP latency differences and **(B)** ERP amplitude differences for each study participant. Differences (*Δ*) were calculated as: incorrect–correct.

As shown in Figure 5, *post hoc* linear regression analysis showed that the relation between latency differences and test scores was not significant, *t* (10) = 1.46, *p* = 0.161, *d* = 0.44, but indicated a positive overall trend. In contrast, the relationship between ERP amplitude differences and *Phase III* test scores was significant, *t* (10) = −3.14, *p* = 0.005, *d* = −0.95, indicating a decrease in mean score of 2.3 percent (95% CI: [−3.88, −0.745]) per 𝜇V increase in amplitude difference for the cohort. Finally, neither the number of *Phase II* trials nor the total number of *Phase I* and *Phase II* trials was a significant mediator in either model of ERP latency or amplitude differences.

## Discussion

The primary objective of this study was to examine feedback-related ERP waveforms during the establishment of conditional discriminations in a matching-to-sample (MTS) training paradigm. To our knowledge, this is the first study to use EEG to measure covert neural responses during the learning phase of conditional discriminations. Our main findings revealed significant paired differences in ERP amplitude and latency between incorrect and correct responses. Additionally, greater ERP amplitude during the establishment of conditional discriminations was inversely related to the formation of equivalence classes. Together, these findings provide novel evidence that feedback-related ERPs are sensitive to early stages of conditional discrimination learning and how they relate to later equivalence class formation.

ERP latency is generally interpreted as reflective of the timing of specific cognitive operations, such as stimulus evaluation or response selection, whereas ERP amplitude reflects the degree of neural resource allocation (Penny et al., 2002). Accordingly, our results suggest that incorrect trials elicited delayed and more resource-intensive processing, indicative of increased cortical recruitment following incorrect trials. This aligns with previous work showing enhanced neural engagement after erroneous decision-making (Bahramali et al., 1998; Hoekzema et al., 2010).

Across participants, ERP activity was localized to frontal electrodes, consistent with the well-established role of the frontal cortex in higher-order functions such as attention, working memory, decision-making, and performance monitoring (Funahashi, 2017). While ERN and FRN are thought to originate primarily from the anterior cingulate cortex (Holroyd and Coles, 2002; Orr and Hester, 2012), the present frontal scalp distribution is compatible with engagement of prefrontal systems involved in strategic learning and cognitive control.

As mentioned, ERN (Falkenstein et al., 1990) and FRN (Miltner et al., 1997) have been elicited by erroneous responses and incorrect feedback. Another potential waveform, a positive feedback-related P300, has been associated with reward processing during associative learning tasks (Paul et al., 2025; Polezzi et al., 2010; Yeung and Sanfey, 2004). This waveform may reflect the extent to which information is motivationally significant or salient (for a review, see Nieuwenhuis et al., 2005). Studies have shown that the P300 amplitude varies with the motivational significance of feedback information (Wu and Zhou, 2009) and is increased in individuals who attribute more meaning to trial feedback (de Bruijn et al., 2004). A single positive deflection was observed between ~154–953 ms following feedback onset in the current study. Peaks were operationally defined as local maxima within this latency window. While the waveform characteristics resemble a feedback-related P300, the extended latency range and consistently frontal distribution raise interpretive uncertainty. It is therefore also plausible that aspects of FRN/ERN contributed to the signal, as both are typically frontocentral and associated with error processing. Given this ambiguity, we interpret the waveform conservatively as a feedback-related ERP component with possible overlap across established families (ERN, FRN, P300). This underscores the need for future studies to employ additional component classification strategies, such as independent component analysis or source localization, to clarify functional origins. Also, exploring different types of programmed consequences (e.g., varying reinforcement value) during conditional discrimination training may offer valuable opportunities to investigate how motivational salience shapes electrocortical dynamics.

Furthermore, exploring the effect of small systematic changes in the experimental paradigm will improve the interpretation of the findings. In the current study, the ERP was locked to the participant’s selection response to one of the comparison stimuli, resulting in the immediate presentation of the programmed consequences. These consequences were presented for 1,000 ms and 500 ms ITI. The timing of the programmed consequences in studies examining the effect of feedback on ERPs varies, often in accordance with the experimental paradigm used. For example, in a time estimation task by Miltner et al. (1997), participants had to emit a response 1,000 ms after a cue was presented. Then, 600 ms elapsed before the feedback was presented. In Pornpattananangkul and Nusslock (2015) the interval from the cue to the guessing response was 3,500 ms, with a 2000 ms interval from the response until the feedback was presented. For the current study, the baseline interval (−200 to 0 ms relative to feedback onset) may have coincided with participants’ response execution and motor preparation. This introduces the possibility that task-related cortical activity may contaminate the baseline, particularly in frontal channels. The amplitude values reported here may reflect a combination of feedback-locked and pre-response activity, a methodological limitation that should be considered when interpreting the results. Addressing this limitation, the current study presents an experimental paradigm that allows for the systematic exploration of the effect of procedural changes on ERPs, thereby enhancing our understanding of the processes involved in learning.

Another important observation was that although ERP triggers were implemented for both *Phase I* and *Phase II* of this study, very few incorrect responses were produced during *Phase II*; indeed, by the final block—when programmed consequences were almost fully faded—no incorrect epochs were available for analysis. For this reason, feedback-related ERP analyses were restricted to *Phase I* data. In that regard, there are two issues that should be addressed. First, the MTS paradigm was designed so that each participant would master the baseline conditional discriminations before proceeding to the next phase of the study. As such, the number of training trials was dependent on the participant’s learning history, rather than a predefined number of “correct” or “incorrect” responses. This approach highlights each participant’s learning process, but at the same time, it precludes control over the number of “incorrect” versus “correct” stimulus presentations. As noted by Bortoloti et al. (2013) and do Espírito-Santo et al. (2020), the different number of programmed consequences during the acquisition phase can influence participants responses in MTS tasks (e.g., accuracy and likelihood of stimulus class formation), and ERPs under investigation (e.g., the N400). Therefore, futures studies may adjust the number of training trials to explore their effect on the ERPs during the establishment of conditional discriminations as well. Second, future studies could incorporate trial-level trigger codes across both correct and incorrect trials, allowing for the examination of whether ERP latencies and amplitudes systematically evolve across training phases. Such data could provide valuable additional insight into the temporal dynamics of cortical recruitment during the transition from initial acquisition to consolidation of conditional discriminations.

Likewise, because this study employed a 200 ms pre-stimulus baseline and 800 ms post-stimulus window—with feedback presented immediately following the selection response—the relatively late peaks observed may have been influenced by heterogeneity in response times. Unfortunately, mean reaction times (RTs) were not recorded in the present study. Future work could incorporate RT tracking, as variability in stimulus–response-feedback timing may critically shape the interpretation of feedback-related ERPs.

The interdisciplinary approach in the current study is noteworthy. Combining experimental analysis of behavior with neurophysiological measures is of both conceptual and applied interest (e.g., Donahoe, 2017; Ortu and Vaidya, 2013; Schlinger, 2015). The analyses in the current study provide new evidence of the cortical neurodynamics during the establishment of conditional discriminations, illustrating correlations between observable behavior and otherwise unobservable neural processes. From a behavior-analytic perspective, understanding the variables that influence the establishment of conditional discrimination may enhance the application of this procedure with different populations. As mentioned, the conditional discrimination is a very well-known procedure within behavior analysis (Cihon et al., 2023; Sidman, 1994; Sidman and Tailby, 1982). It is based on principles of operant learning and is frequently used in both experimental and applied settings. As such, adding EEG as an additional measure provides a path for continued systematic research, which has been called for (e.g., San Martín, 2012). Also, since the conditional discrimination has been suggested to be useful for both understanding changes in neurocognitive disorders (Arntzen and Steingrimsdottir, 2017; Sidman, 2013) and as potential procedure to maintaining stimulus classes in older adults with neurocognitive disorders (Brogård-Antonsen and Arntzen, 2019), or individuals with acquired brain injury (e.g., Cowley et al., 1992) incorporating EEG is especially valuable to learn more about co-occurring covert processes when using this procedure in applied setting. This is not only of conceptual or applied interest but also touches upon an ethical aspect when working with vulnerable populations, as it is important that procedures used in the clinical setting are based on scientific knowledge. As such, combining theoretical approaches may lead to better clinical practice. This relates to studies showing that associative learning deficits are observed in several clinical populations, including individuals with autism spectrum disorder (Desaunay et al., 2020), Alzheimer’s disease (Boespflug et al., 2014; Lee et al., 2013), and traumatic brain injury (Fortin et al., 2021). Identifying ERP signatures of impaired feedback processing could contribute to early detection of neurocognitive alterations and inform intervention strategies (e.g., Arntzen and Steingrimsdottir, 2017; Brogård-Antonsen and Arntzen, 2019; Cowley et al., 1992; Harker and Connolly, 2007). Generally, further investigation of EEG measures may help evaluate the integrity of associative learning systems in these vulnerable patient populations.

A limitation of this study includes its modest sample size, determined by convenience (n = 11), which, although sufficient to detect group-level effects, limited our ability to explore the influence of covariates such as age, sex, or education (Yerlikaya et al., 2022). Also, with such a modest sample size, the likelihood of false positives may increase (Button et al., 2013). Methodological differences from prior ERP studies—including stimulus modality and task structure—may also affect generalizability (San Martín, 2012). Furthermore, 5 of the 16 recruited participants (31%) did not complete the protocol due to discontinuation or procedural interruptions. As such, the ERP data included were derived from a subsample of individuals who were particularly able to tolerate the cognitively and physically demanding nature of extended MTS training and EEG recording. This raises the possibility that the final sample reflected participants with higher-than-average attentional control, cognitive stamina, or task persistence, which may limit the external validity of the findings. Taken together, to address these concerns, replication with a larger group of participants is warranted.

In conclusion, to our knowledge, this study provides the first evidence of feedback-related ERP differences during the establishment of conditional discriminations in an MTS format, demonstrating their sensitivity to correct versus incorrect programmed consequences and how they relate to later equivalence class formation. These findings underscore the value of integrating EEG with behavior-analytic methods to illuminate covert neural processes underlying associative learning.

## Data Availability

The raw data supporting the conclusions of this article will be made available by the authors, without undue reservation.

## Footnotes

1
https://www.psychopy.org/
